# Modeling of Statistical Variation Effects on DRAM Sense Amplifier Offset Voltage

**DOI:** 10.3390/mi12101145

**Published:** 2021-09-23

**Authors:** Kyung Min Koo, Woo Young Chung, Sang Yi Lee, Gyu Han Yoon, Woo Young Choi

**Affiliations:** 1 Department of Electronics Engineering, Sogang University, Seoul 04107, Korea; kyung1573@naver.com (K.M.K.); ghyoon@sogang.ac.kr (G.H.Y.); 2 Department of DRAM Sensing & Advanced Analysis, SK Hynix, Icheon 17336, Korea; wooyoung.chung@sk.com (W.Y.C.); sangyi.lee@sk.com (S.Y.L.)

**Keywords:** dynamic random access memory, sense amplifier, sensitivity, offset voltage, variation, threshold voltage mismatch

## Abstract

With the downscaling in device sizes, process-induced parameter variation has emerged as one of the most serious problems. In particular, the parameter fluctuation of the dynamic random access memory (DRAM) sense amplifiers causes an offset voltage, leading to sensing failure. Previous studies indicate that the threshold voltage mismatch between the paired transistors of a sense amplifier is the most critical factor. In this study, virtual wafers were generated, including statistical V_T_ variation. Then, we numerically investigate the prediction accuracy and reliability of the offset voltage of DRAM wafers using test point measurement for the first time. We expect that this study will be helpful in strengthening the in-line controllability of wafers to secure the DRAM sensing margin.

## 1. Introduction

**Figure 1:**
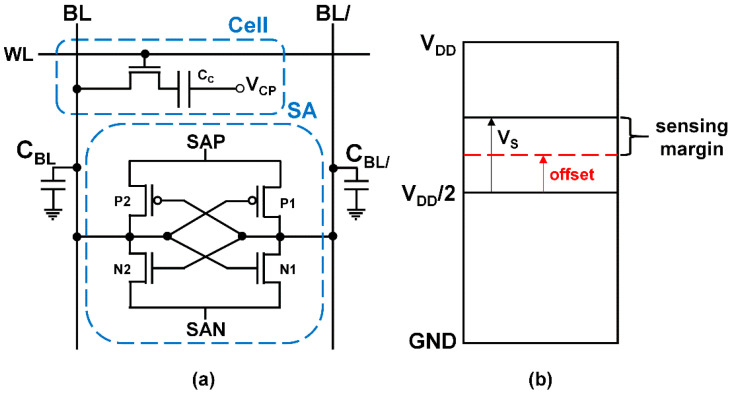
(**a**) Schematic illustration of DRAM, which consists of a cell and an SA. (**b**) Voltage diagram of the sensing margin. *V*_S_ must be larger than the relevant offset voltage for correct sensing; otherwise, the opposite data value will be read.

Artificial intelligence (AI) and 5G networks are emerging as major topics in information technology (IT). These applications require high-density and low-power memory. Dynamic random access memory (DRAM) can play an important role, owing to its fast switching speed, low bit cost, and high memory density [1]. Figure 1a shows a schematic of a DRAM cell connected to a sense amplifier (SA). The cell part consists of a cell transistor acting as a switch and a cell capacitor storing charge. The cell plate voltage (*V*_CP_) is connected to half-*V*_DD_ to optimize the leakage current of the cell capacitor. An SA is a complementary metal-oxide-semiconductor (CMOS) latch using the half-*V*_DD_ precharge-sensing method [2]. The basic operating mechanism of read ‘1’ is as follows: In the standby mode, the bit-line (BL) pair voltage is precharged to half-*V*_DD_. When the word-line (WL) voltage is raised to the ‘high’ level, the charge stored in the cell capacitors is transferred to the BLs. Then, the BL voltage deviates from half-*V*_DD_, and a small voltage difference (*V*_S_) between BL and BL/ is generated as follows:

(1) $$V_{S}=\frac{V_{DD}/2}{1+C_{BL}/C_{C}}$$

where *C*_BL_ and *C*_C_ are the BL capacitance and the cell capacitance, respectively. With SA activation, the BL voltage is amplified up to *V*_DD_ by the positive feedback of the latched inverters of the SA, and data ‘1’ is read. However, accurate data sensing is feasible only when *V*_S_ exceeds the SA offset voltage, as shown in Figure 1b. Therefore, if *V*_S_ becomes lower than the SA offset voltage, the sensed data read inaccurately.

With advances in DRAM generation, both *V*_DD_ and *C*_C_ are being scaled down [3,4], thereby reducing the *V*_S_. This reduction in cell transistor size also increases the threshold voltage (*V*_T_) variation [5], resulting in a larger offset voltage. Thus, the offset voltage characterization becomes more important in SA sensitivity improvement.

R. Kraus et al. derived the offset voltage theoretically by using differential equations, confirming that simultaneous sensing was more advantageous than one-delayed sensing [6]. R. Sarpeshkar et al. derived a rigorous formula considering various parameter mismatches and showed a good agreement compared with HSPICE simulation results [7]. Among the various sources of offset voltage, including parasitic capacitance and *β* and *V*_T_ variation, *V*_T_ mismatch (Δ*V*_T_) of the SA transistors has been considered as the most dominant factor [7,8,9]. At the chip (die) level, offset voltage is calculated by statistically measuring many SAs in a die. S. M. Kim et al. [10] investigated the SA sensing failure percentage in a die according to the *V*_DD_, the *V*_T_ variation, and the channel width ratio of NMOS and PMOS using a Monte Carlo simulation. Because the *V*_T_ in a die follows the Gaussian distribution [11], the offset voltage also is assumed to follow the same distribution. Thus, the *V*_T_ standard deviation can be a good indicator of estimating the die offset voltage. S. H. Woo et al. [12] proposed an offset voltage variance estimation model considering the secondary effects such as drain-induced barrier lowering (DIBL), differential charge injection (DCI), and stack effects. Y. Li et al. [13] investigated the DRAM-SA mismatch analytically using small-signal analysis and optimized the result to obtain the minimum offset voltage variance. Then, they derived a linear model considering the sensing delay of SAs and confirmed that simultaneous sensing minimized the die level offset voltage. However, to the best of our knowledge, no study has been attempted to cover the offset voltage at a wafer level.

In this study, virtual wafers are generated based on the global and local variation theory, and the statistical simulation results of the offset voltage distribution at the die and wafer levels are obtained using test point measurement, which is widely used for wafer property identification. Finally, we numerically analyze the offset voltage prediction accuracy and probability of DRAM wafers for the first time. We expect that this study can be used as important information in the DRAM process line and consequently help secure the sensing margin of the DRAM.

The remainder of this study is organized as follows: in Section 2, the *V*_T_ variation theory is explained. Then, the assumption of generating virtual wafers and the methodology of extracting data is described in Section 3. Finally, the results are discussed in Section 4.

## 2. *V*_T_ Variation

With the reduction in device sizes, the device parameter fluctuations and short-channel effects need a thorough investigation [14]. It is widely known that the process variations, including random dopant fluctuation (RDF), line edge roughness (LER), and work function variation (WFV), affect nonuniform *V*_T_ distribution [5,15,16,17]. Especially, process variation was classified into two categories: global and local variations [18]. 

**Figure 2:**
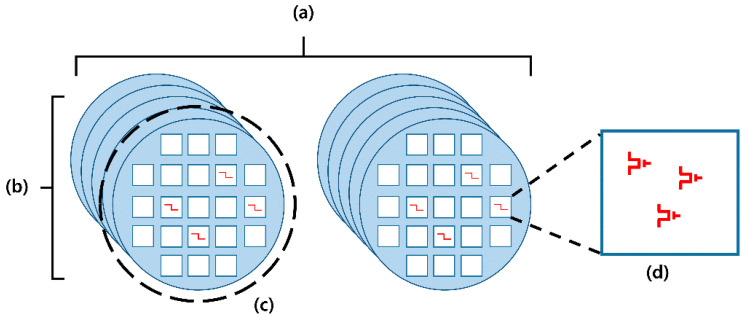
Classification of variations. Global variations include (**a**) lot-to-lot variation (LTLV), (**b**) wafer-to-wafer variation (WTWV), and (**c**) die-to-die variation (DTDV). Local variation means (**d**) within-die variation (WIDV).

**Figure 3:**
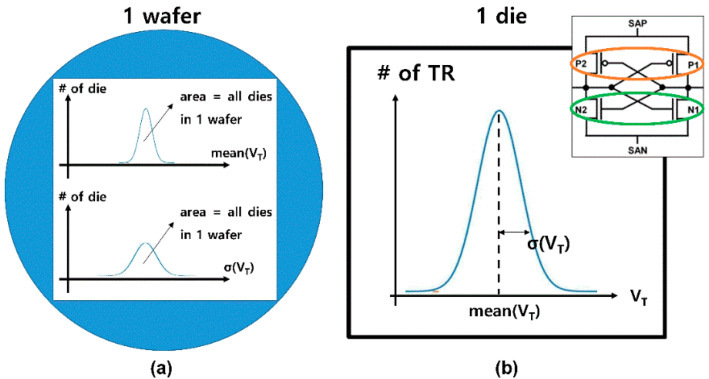
DTDV and WIDV in this work. (**a**) DTDV of mean(*V*_T_)s and σ(*V*_T_)s are assumed to follow the Gaussian distribution. (**b**) WIDV exists in a normal distribution. Transistor pairs ({N1, N2} or {P1, P2}) have same distribution properties.

First, the global variation includes lot-to-lot variation (LTLV, Figure 2a), wafer-to-wafer variation (WTWV, Figure 2b), and die-to-die variation (DTDV, Figure 2c). Because global variation is location-dependent, it can be characterized by wafer maps. For a simple and concise discussion, a Gaussian distribution was applied to global variation, as shown in Figure 3a.

Second, the local variation includes the within-die variation (WIDV) shown in Figure 2d. Within a die, *V*_T_ follows a Gaussian distribution with a certain mean (mean(*V*_T_)) and standard deviation (σ(*V*_T_)) independent of location (random distribution). In this study, *V*_T_ of the SA’s transistors is assumed to follow a Gaussian distribution and the *V*_T_s of the transistor pair sharing the same SA follow the same Gaussian distribution, as shown in Figure 3b.

## 3. Simulation Methodology

First, we modeled the offset voltage distribution of one die by referring to [13], which statistically investigated the offset voltage using small-signal analysis and showed good agreement with simulation results. According to [13], the variance of offset voltage of simultaneously latched CMOS SAs in one die is expressed as follows:

(2) $$\sigma^{2}\left(V_{OS}\right)=\frac{2\sigma^{2}\left(\Delta V_{TP}\right)\sigma^{2}\left(\Delta V_{TN}\right)}{\sigma^{2}\left(\Delta V_{TP}\right)+m\sigma^{2}\left(\Delta V_{TN}\right)}$$

where *V*_OS_ is the offset voltage of one SA in a die. Moreover, Δ*V*_TN_ and Δ*V*_TP_ represent the *V*_T_ mismatch of paired NMOS and PMOS in a SA, respectively. The constant *m* is expressed as follows:

(3) $$m=\frac{V_{DD}-\left(2+(1/\alpha )\right)V_{TN}+\left(1/\alpha \right)\left|V_{TP}\right|}{V_{DD}-|V_{TP}|-V_{TN}}$$

where *α* is expressed in terms of *V*_DD_ and *V*_T_:

(4) $$\alpha =\frac{V_{DD}-2\left|V_{TP}\right|}{V_{DD}-2V_{TN}}$$

In this article, *V*_DD_ is assumed to be 1.2 V. In addition, the average *V*_TN_ and *V*_TP_ are assumed to be 0.423 V and −0.365 V, respectively. Accordingly, *α* and *m* are calculated as 1.328 and 0.7534, respectively.

As the offset voltage of a die (*V*_OS,die_) is statistically defined, we choose the 4σ value of the single SA offset voltage distribution, which is calculated by using Equation (5):

(5) $$V_{OS,die}=4\sigma \left(V_{OS}\right)$$

**Figure 4:**
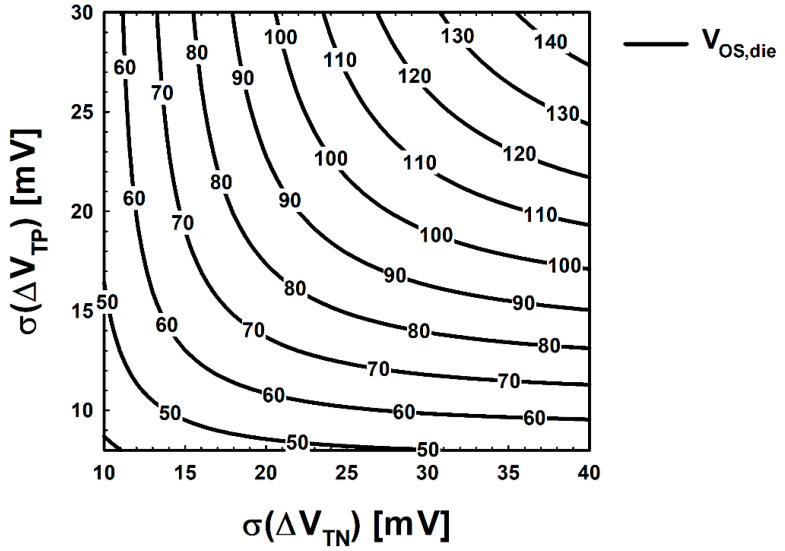
Contours of *V*_OS,die_ used in this study.

Then, the offset voltage map according to σ(Δ*V*_TN_) and σ(Δ*V*_TP_) is plotted as shown in Figure 4. It is observed that *V*_OS,die_ increases as σ(Δ*V*_TN_) or σ(Δ*V*_TP_) increases.

**Figure 5:**
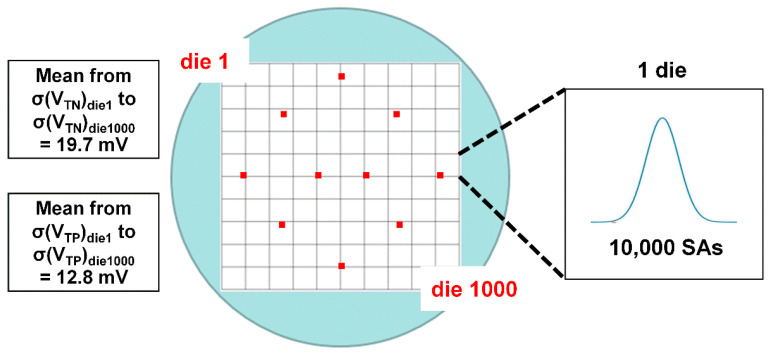
Assumption of virtual wafer in this work. One wafer includes 1000 DRAM dies. It is assumed that the average σ(*V*_TN_) and σ(*V*_TP_) of dies in wafer are 19.7 mV and 12.8 mV, respectively. There are 10,000 SAs in 1 DRAM die, and their characteristics follow the Gaussian distribution. Red dots on a wafer indicate the 10 test points.

Next, we made a virtual wafer including 1000 DRAM dies. Additionally, for a simple and concise discussion, we assumed that each DRAM die had 10,000 SAs, since a desirable Gaussian distribution can be formed with just that number. As a result, the *V*_T_s of SAs in a die follow a Gaussian distribution. Because it has been proven by previous studies that the major factor that affects the offset voltage is Δ*V*_T_, we considered only σ(*V*_T_) and σ(Δ*V*_T_) for the concise discussion. Thus, the average values of σ(*V*_TN_) and σ(*V*_TP_) of dies in a virtual wafer are assumed to be 19.7 mV and 12.8 mV, respectively [11], which is shown in Figure 5. Then, to calculate the average offset voltage of dies in a wafer, we can apply a simple statistical equation to derive σ(Δ*V*_T_) from σ(*V*_T_). Since we assume WIDV as a random variation, the *V*_T_ of each SA transistor pair is independent of each other. Therefore, the relationship between the variance of Δ*V*_T_ (σ^2^(Δ*V*_T_)) and that of *V*_T_ (σ^2^(*V*_T_)) is given by the following equation [14]:

(6) $$\sigma^{2}\left(V_{T1}-V_{T2}\right)=\sigma^{2}\left(\Delta V_{T}\right)=2\sigma^{2}\left(V_{T}\right)$$

Accordingly, the σ(Δ*V*_T_) can be expressed as follows:

(7) $$\sigma \left(\Delta V_{T}\right)=\sqrt{2}\sigma \left(V_{T}\right)$$

As a consequence, when σ(*V*_TN_) is 19.7 mV, σ(Δ*V*_TN_) is calculated as 27.86 mV, and when σ(*V*_TP_) is 12.8 mV, σ(Δ*V*_TP_) is calculated as 18.01 mV, respectively. From σ(Δ*V*_TN_), σ(Δ*V*_TP_) and Equation (2), the average offset voltage of dies in a wafer is analytically calculated as 94.44 mV.

Here, we explain the offset voltage prediction method. The average offset voltage of dies in a wafer is predicted as follows. First, 10 test points that can represent the whole wafer are selected, as shown in Figure 5. Then, Δ*V*_TN_ and Δ*V*_TP_ are extracted from that point. Afterward, σ(Δ*V*_TN_) and σ(Δ*V*_TP_) are calculated from these 10 Δ*V*_TN_ and Δ*V*_TP_. Then, these values would be used to predict the offset voltage. The results of prediction and analysis of accuracy will be discussed in the latter part of this paper.

## 4. Results and Discussion

**Figure 6:**
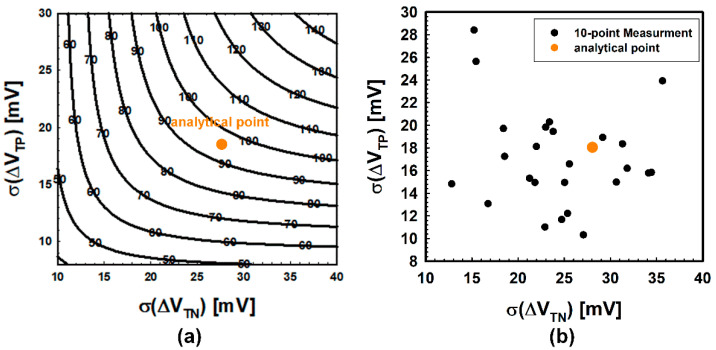
Offset voltage contours and prediction results. (**a**) Offset voltage contours (black line) and analytical point (orange dot). At the analytical point, the offset voltage is 94.44 mV. (**b**) Analytical point (orange dot) and predicted points (black dots) using 10-point measurements. The extracted data are distributed without a trend.

**Figure 7:**
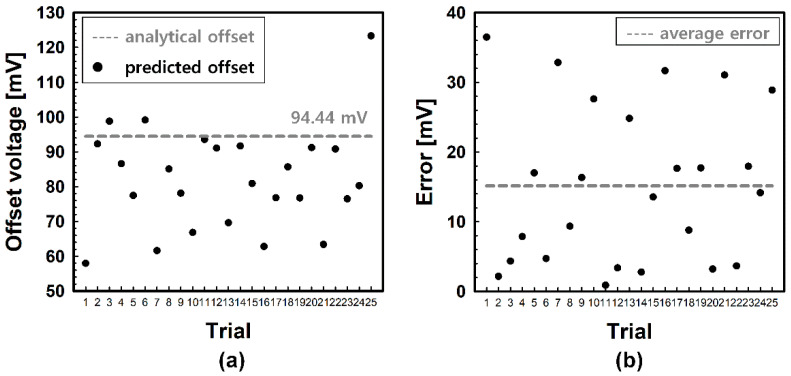
Predicted offset voltages and errors of 25 wafers for further investigation. (**a**) Analytical offset voltage and predicted offset voltages. (**b**) Calculated errors between analytical offset voltage and predicted offset voltages.

For intuitive comparison, simulation results are pointed with an analytical point which is shown in Figure 6. The orange point in Figure 6a,b indicates the analytical point (27.86 mV, 18.01 mV), and the offset voltage at this analytical point is 94.44 mV. Black points in Figure 6b indicate the predicted points using the 10-point measurement. Each black point in Figure 6b was extracted from one of the 25 identical wafers. As shown in Figure 6b, the 10-point prediction is not trending and has a wide distribution, which is estimated to be an insufficient number of samples, which were not enough to accurately predict the offset voltage of a wafer. Furthermore, the maximum distance in Figure 6b between the analytical point and predicted a point is calculated as 24.58. However, since the distance from the analytical point does not have a linear correlation with the error (see Figure 6a), we calculate the error between the offset voltage at the analytical point and at the predicted point to clarify the accuracy of the prediction. Figure 7 shows the predicted offset voltage (Figure 7a) and error (Figure 7b) of the 25 wafers. As shown in Figure 7, the overall predicted offset voltage is distributed far from the analytical value, and the maximum error and the average error are estimated to be 38 mV and 15 mV, respectively. The ratio of the average error, 15 mV, to the analytical offset voltage is a somewhat large value, which is equivalent to 16% and needs to be decreased for more accurate prediction.

**Figure 8:**
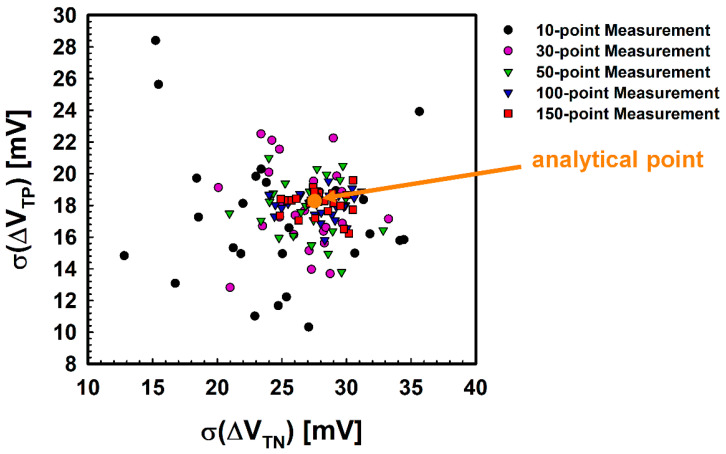
Analytical point and predicted points according to the number of test points. As the number of test points increases, the predicted points concentrate around the analytical point.

**Figure 9:**
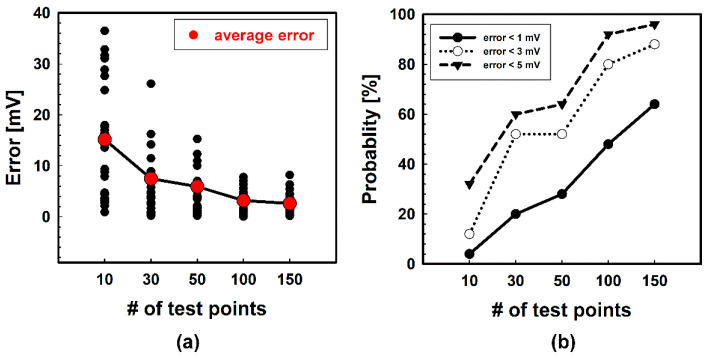
Errors and prediction probability of each number of test points. (**a**) Errors between the analytical point and the predicted points according to the various numbers of test points. (**b**) Prediction probability according to the various numbers of test points and allowable error.

Hence, we increased the number of test points to strengthen the prediction accuracy and verify how much the accuracy is improved according to the number of test points. Besides 10-points measurements, 30, 50, 100, and 150 points were selected, and data were extracted in the same way. Figure 8 shows the results with various numbers of test points. As expected, it appears that the predicted points are moving toward the analytical point as the number of test points increases to 100 points. However, there seems to be little difference between the prediction results of 100-points measurements and 150-points measurements. To further analyze the improvement in prediction accuracy, the error and the corresponding probability plot were also calculated. As the number of test points increases, the distribution of error is diminished, and the average error is reduced, as shown in Figure 9a. Notably, the average error is reduced below 3 mV when the number of test points is 100, and further improvement is minimal when the number grows from 100 points to 150 points. Likewise, the prediction probability is also enhanced as the number of test points increases, which is described in Figure 9b. Of course, the smaller the allowable error, the lower this probability is. However, when the allowable error is 5 mV, it is confirmed that the 100-point measurements show more than 90% reliability. Given these facts, it is estimated that at least 100-point measurements will be needed to reliably predict the overall offset voltage of the wafer by measuring the test points.

Then, we made other types of virtual wafers to examine how the prediction accuracy changes with regard to variation properties. The aforementioned wafer was named ‘w0’, and the rest of the wafers (from ‘w1’ to ‘w6’) were set by increasing and decreasing the average value of σ(*V*_TN_) and σ(*V*_TP_) of dies in wafer ‘w0’ by 20%, respectively. The variation properties and analytical offset voltages of wafers are summarized in Table 1. For an accurate comparison, the number of test points is chosen as 100, and the simulation was performed in the same way.

**Figure 10:**
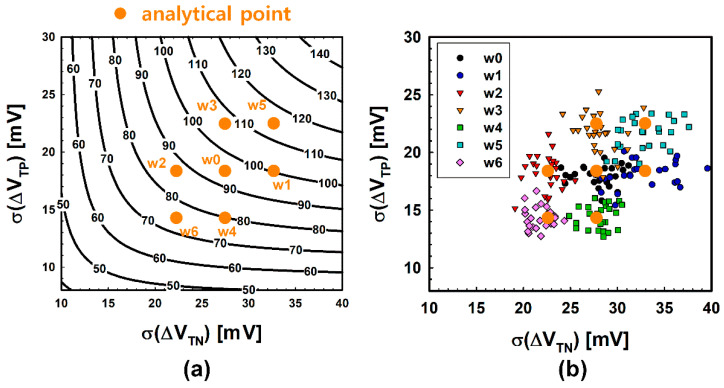
Offset voltage contours and predicted points of each wafer. The orange points represent the analytical offset voltages of each wafer. (**a**) Offset voltage contours. The analytical offset voltage of each point is: ‘w0’ = 94.44 mV, ‘w1’ = 100.1 mV, ‘w2’= 86.14 mV, ‘w3’ = 105.3 mV, ‘w4’ = 80.96 mV, ‘w5’ = 113.33 mV, ‘w6’ = 75.55 mV. (**b**) Predicted points of each wafer. A total of 100 test points are selected in 1 wafer for a reliable prediction.

**Figure 11:**
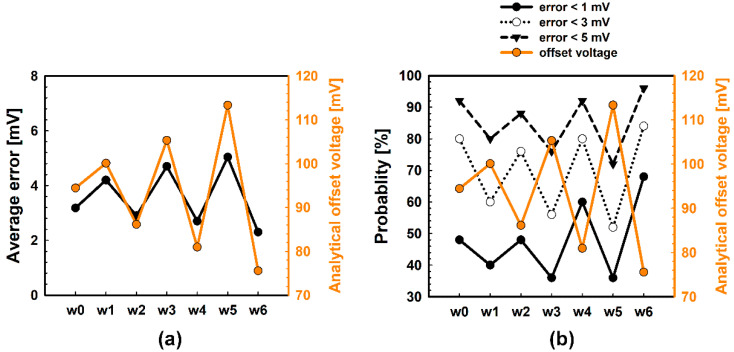
Average errors and prediction probability of each wafer. (**a**) The greater the variation in a wafer, the greater the average error. (**b**) Thus, the wafer with the largest variation has the worst predictive accuracy.

Figure 10 shows the result of the simulation. In Figure 10a, analytical points of each wafer are marked on the offset voltage contours. Since 100 points were measured to investigate the desirable accuracy, a number of relevant predicted points are placed near each analytical point, as shown in Figure 10b. Then, a quantitative analysis of the error is described in Figure 11. Interestingly, it is confirmed that the average error has a positive correlation with the analytical offset voltage (see Figure 11a). In other words, the wafer with the largest variation has a larger prediction error. This is because the greater the population variance is, the more the consistency of the sample variances decreases. For this reason, regarding the prediction probability, the wafer with the largest variation is more likely to make a poor prediction. Specifically, as shown in Figure 11b, when the allowable error is 3 mV, the prediction probability falls to around 50% at wafer ‘w5′, which has the greatest variation.

## 5. Conclusions

Owing to the increase in demand for DRAM and the scaling of device technology nodes, the offset voltage characteristics of the DRAM SA are becoming increasingly important to design a sensitive SA. In this study, we numerically analyzed the prediction accuracy and reliability of the offset voltage of DRAM wafers using test point measurement for the first time. We created a virtual wafer and then compared the analytical offset voltage of the wafer with the predicted value obtained through Δ*V*_T_ measurement at the test points. With regard to the number of test points, 100-point measurements show more than 90% reliability when the allowable error is 5 mV. Additionally, it is confirmed that the predictive reliability of wafers with small variations is higher. We expect that this study can be used as important information in the DRAM process line, and it will be helpful in strengthening the in-line controllability of wafers to secure the DRAM sensing margin.

## Figures and Tables

**Table 1:** Variation characteristics and analytical offset voltages of each wafer.

Wafer	w0	w1	w2	w3	w4	w5	w6
Average σ(*V*_TN_) of dies (mV)	19.7	23.64	15.76	19.7	19.7	23.64	15.76
Average σ(*V*_TP_) of dies (mV)	12.8	12.8	12.8	15.36	10.24	15.36	10.24
Analytical offset voltage (mV)	94.44	100.1	86.14	105.3	80.96	113.33	75.55

## Data Availability

Not applicable.
